# Causal effects of B vitamins and homocysteine on obesity and musculoskeletal diseases: A Mendelian randomization study

**DOI:** 10.3389/fnut.2022.1048122

**Published:** 2022-11-24

**Authors:** Liwan Fu, Yuquan Wang, Yue-Qing Hu

**Affiliations:** ^1^Center for Non-Communicable Disease Management, Beijing Children's Hospital, Capital Medical University, National Center for Children's Health, Beijing, China; ^2^State Key Laboratory of Genetic Engineering, Human Phenome Institute, Institute of Biostatistics, School of Life Sciences, Fudan University, Shanghai, China; ^3^Shanghai Center for Mathematical Sciences, Fudan University, Shanghai, China

**Keywords:** homocysteine, musculoskeletal diseases, obesity, B vitamins, Mendelian randomization

## Abstract

**Objectives:**

Although homocysteine (Hcy) increases the risk of cardiovascular diseases, its effects on obesity and musculoskeletal diseases remain unclear. We performed a Mendelian randomization study to estimate the associations between Hcy and B vitamin concentrations and their effects on obesity and musculoskeletal-relevant diseases in the general population.

**Methods:**

We selected independent single nucleotide polymorphisms of Hcy (*n* = 44,147), vitamin B12 (*n* = 45,576), vitamin B6 (*n* = 1864), and folate (*n* = 37,465) at the genome-wide significance level as instruments and applied them to the studies of summary-level data for fat and musculoskeletal phenotypes from the UK Biobank study (*n* = 331,117), the FinnGen consortium (*n* = 218,792), and other consortia. Two-sample Mendelian randomization (MR) approaches were utilized in this study. The inverse variance weighting (IVW) was adopted as the main analysis. MR-PRESSO, MR-Egger, the weighted median estimate, bidirectional MR, and multivariable MR were performed as sensitivity methods.

**Results:**

Higher Hcy concentrations were robustly associated with an increased risk of knee osteoarthritis [odds ratio (OR) 1.119; 95% confidence interval (CI) 1.032–1.214; *P* = 0.007], hospital-diagnosed osteoarthritis (OR 1.178; 95% CI 1.012–1.37; *P* = 0.034), osteoporosis with pathological fracture (OR 1.597; 95% CI 1.036–2.46; *P* = 0.034), and soft tissue disorder (OR 1.069; 95% CI 1.001–1.141; *P* = 0.045) *via* an inverse variance weighting method and other MR approaches. Higher vitamin B12 levels were robustly associated with decreased body fat percentage and its subtypes (all *P* < 0.05). Bidirectional analyses showed no reverse causation. Multivariable MR analyses and other sensitivity analyses showed directionally similar results.

**Conclusions:**

There exist significant causal effects of vitamin B12 in the serum and Hcy in the blood on fat and musculoskeletal diseases, respectively. These findings may have an important insight into the pathogenesis of obesity and musculoskeletal diseases and other possible future therapies.

## Introduction

Musculoskeletal diseases are common and have an increased global prevalence (1). In addition to being a significant contributor to the global illness burden, they are also considered driving forces behind the Bone and Joint Decade (2). Recent evidence suggests that metabolic complications, such as lean mass loss in people with obesity and osteoporosis, can increase the risk of musculoskeletal diseases (3). However, novel bone and nerve regeneration techniques have been developed (4, 5). Low lean mass and quality with elevated fat mass were not only correlated with poor physical function (6) but also could lead to an elevation in the risk for serious musculoskeletal diseases (7, 8). To some degree, fat mass, lean mass, and musculoskeletal diseases—these three compositions—have very strong physiological connections (6–8).

**Figure 1:**
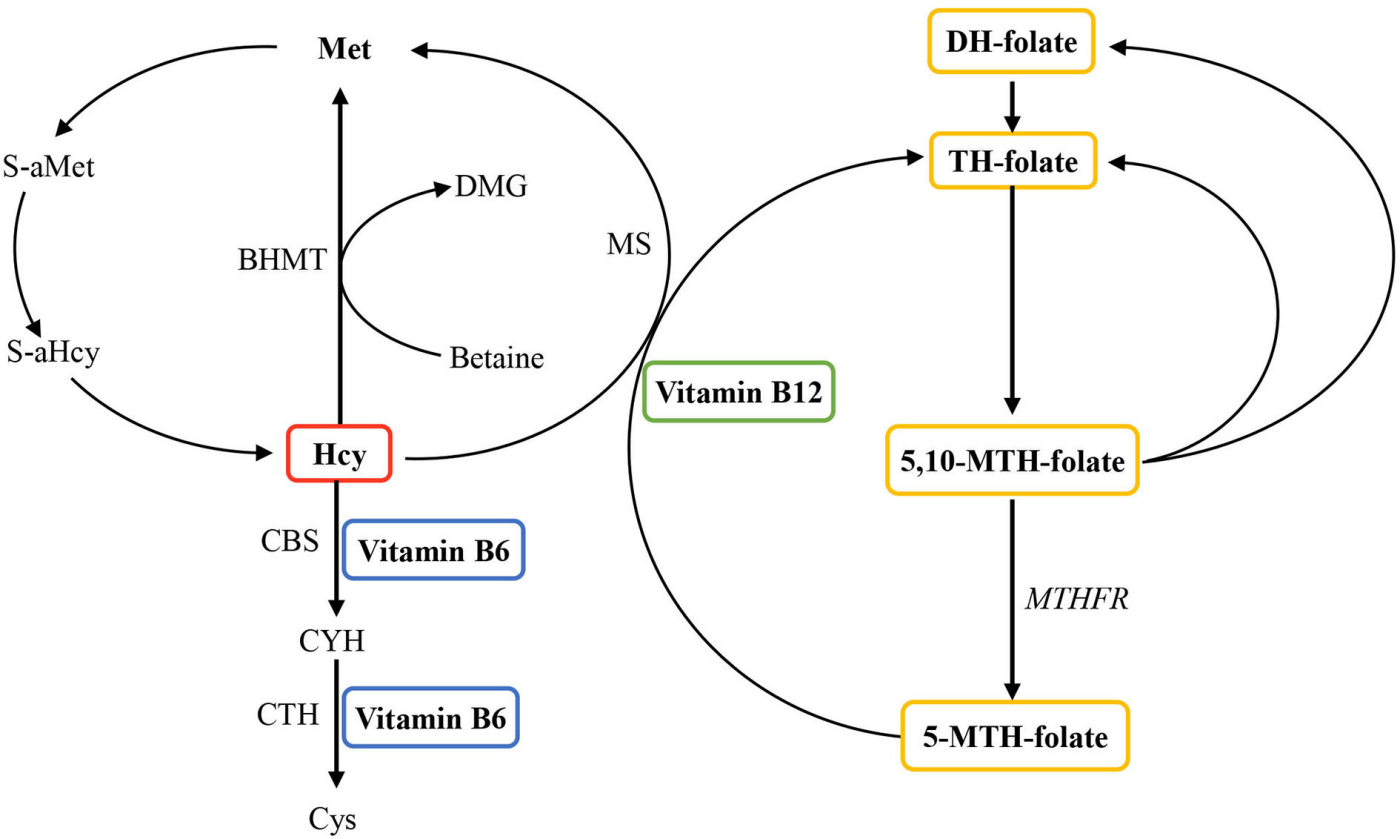
Overview of the role of vitamin B6, vitamin B12, and folate in homocysteine metabolism. Homocysteine is reconverted to methionine by acquiring a methyl group from 5-methyltetrahydrofolate, the positive form of betaine or folate in the remethylation pathway. Irreversible removal of homocysteine happens across the transsulfuration pathway in which homocysteine condenses with serine to form cystathionine. BHMT, betaine homocysteine methyltransferase; CBS, cystathionine-β-synthase; CYH, cystathionine; CTH, cystathionine-gamma-ligase; Cys, cysteine; DH, dihydo; DMG, dimethylglycine; Hcy, homocysteine; Met, methionine; MS, methionine synthase (encoded by the MTR gene); MTH, methylenetetrahydrofolate; MTHFR, methylenetetrahydrofolate reductase; S-aMet, S-adenosylmethionine; S-aHcy, S-adenosylhomocysteine; TH, tetrahydro.

B vitamins, including vitamins B6 and B12, and folate, play a crucial role in the metabolism of homocysteine (Hcy) (Figure 1). A lack of these B vitamins can result in a higher concentration of total Hcy, which has been considered a risk factor and affects cardiovascular diseases (9–11). Previous research has suggested that Hcy levels and folate concentrations impact the increased risk of obesity (12–14). However, the mechanisms concerning these observations remain unclear. There is a great chance that the elevated Hcy levels might affect the development of obesity *via* regulating body fat storage in the process of epigenetic regulation because a strong association exists between Hcy metabolism and DNA methylation and amino acid residues on histones (15–17). Hcy-thiolactone, a product of an error-correcting reaction in protein biosynthesis, is generated in the human body when Hcy is selected in place of methionine by methionyl-tRNA synthetase. It is a chemically reactive thioester metabolite that modifies protein lysine residues in a process called N-homocysteinylation. The modification causes protein damage/aggregation, a hallmark of many diseases, including fat and musculoskeletal diseases (18). Additionally, emerging evidence also indicates the possible role of Hcy in bone and lean mass (19–21). *In vitro* studies have indicated that Hcy could elevate the activity and differentiation of osteoclasts, reduce the quality of lean mass, and then produce the apoptosis of human bone marrow stromal cells by enhancing reactive oxygen species (21). Moreover, Hcy also can decrease bone blood flow, which potentially comes to terms with mechanical bone properties (20). Furthermore, Hcy influences bone tissue formation by interrupting the development of collagen cross-links and decreasing bone blood flow (19, 20). In humans, rigorously high Hcy because of cystathionine-β-synthase deficiency leads to connective tissue abnormalities in most body systems, especially the bones and vasculature (18). High Hcy because of methylenetetrahydrofolate reductase has also been correlated with bone abnormalities in humans (22). Similar connective tissue abnormalities have been found in mice (23). It has been recognized that collagen cross-linking defects might be involved (23).

**Figure 2:**
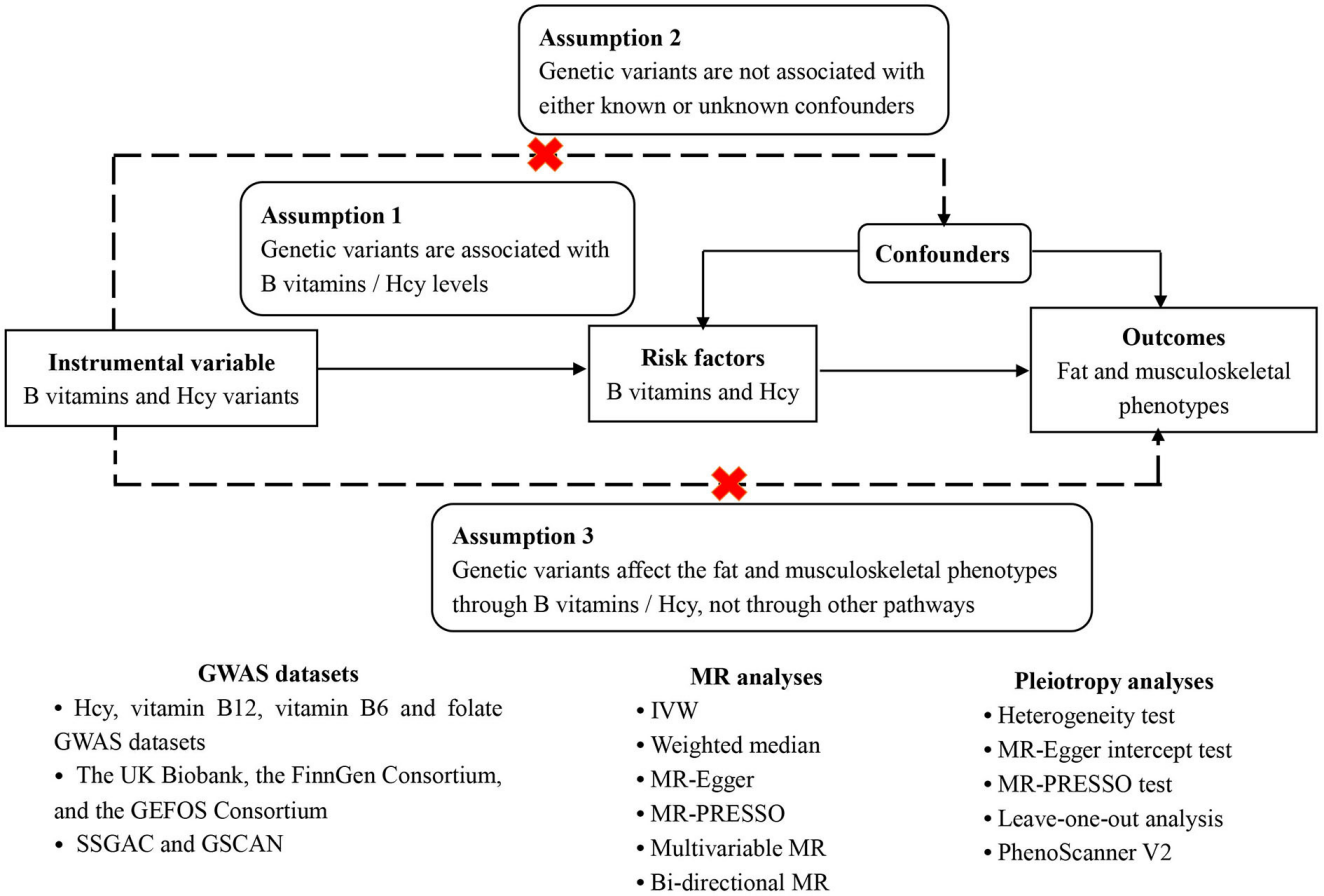
Schematic overview of this MR study design.

The aforementioned evidence shows the biological plausibility that B vitamins and Hcy could contribute to fat and lean mass changes, which would then elevate the risk of musculoskeletal-relevant diseases. However, there exist inconsistent findings about the associations between B vitamins and Hcy with body composition and musculoskeletal diseases in observational studies (12–14, 24–35), necessitating the inference of the causal associations of B vitamins and Hcy with fat percentage, lean mass, and musculoskeletal diseases to offer accurate causal relationships between them. Nowadays, employing the genetic variants as instruments for exposure (e.g., Hcy and vitamin B12), the Mendelian randomization (MR) study could improve causal inference *via* diminishing residual confounding and reverse causation (36). Here, we conducted an MR study to explore the associations of genetically predicted Hcy, vitamins B6 and B12, and folate concentrations with fat and musculoskeletally relevant phenotypes. The specific schematic overview of this MR study design can be found in Figure 2.

## Materials and methods

This is a two-sample MR study employing the publicly available Genome-Wide Association Study (GWAS) data, which should satisfy three assumptions (Figure 2). Firstly, the genetic variants have to be independently associated with the exposures (B vitamins and Hcy), here regarded as the genome-wide significant level (*P* < 5 × 10^−8^) with r^2^ < 0.01. Secondly, other confounders should not affect the association between the genetic variants and the outcomes. Thirdly, the genetic variants influence the outcomes only through their impact on the exposures, i.e., the exclusion restriction assumption (37, 38). This assumption was evaluated by horizontal pleiotropy, in which the variants influence the outcomes directly or *via* other identified causes of outcomes that influence the exposures (39). In addition, bi-directional MR analyses and a multivariable MR model (40) were further employed to test whether B vitamins and Hcy remain considered the true causal factors of fat and musculoskeletal phenotypes, given several confounders. Figure 2 shows the overview diagram of this study's design.

### Ethical approval

This MR study applies public or published data. No original data were required for this study. Ethical approval and informed consent from each subject for every included study in the research can be described in the original publications.

### Genetic predictor of exposures

We selected SNPs that are associated with serum B vitamins or blood homocysteine according to the meta-analyses of GWAS on serum vitamin B12 (*n* = 45,576 subjects) (41), folate (*n* = 37,465 subjects) (41), vitamin B6 (*n* = 1,864 subjects) (42), and blood Hcy (*n* = 44,147 subjects) (43) in European ancestry at the genome-wide significant level (*P* < 5 × 10^−8^) as a genetic predictor of exposures. Based on the 1,000 Genomes European Reference Panel, linkage disequilibrium among SNPs associated with each exposure was evaluated using the PLINK clumping technique. As a result, distinct SNPs without linkage disequilibrium (r^2^ < 0.01) were employed as the instruments (Supplementary Table 1). Therefore, there are 14 independent SNPs for Hcy, 14 for vitamin B12, two for folate, and one SNP for vitamin B6 (Supplementary Table 1). Overall, the SNPs explained 6.0% of the variance for vitamin B12 (41) and Hcy (43), as well as 1.3 and 1.0% of the variance for vitamin B6 (42) and folate (41), respectively.

### Genetic associations with outcomes

Complete GWAS summary data for the outcomes, namely fat percentage and its subtypes (from the UK Biobank) (44), musculoskeletally relevant phenotypes including musculoskeletal system diseases, soft tissue disorders, osteoporosis with pathological fracture, knee osteoarthritis, hip osteoarthritis, and hospital-diagnosed osteoarthritis (data from the UK Biobank and FinnGen Consortium) (44, 45), and lean mass and its subtypes (from the GEFOS Consortium) (46, 47) were acquired from the publicly available online datasets or public GWAS summary data repositories. Detailed descriptions of data sources are exhibited in Table 1. The specific descriptions of diseases and phenotypes can be found in Nealelab (http://www.nealelab.is/uk-biobank), the FinnGen Consortium (https://www.finngen.fi/en/access_results), and the GEFOS Consortium (http://www.gefos.org).

**Table 1:** Information on the genome-wide association studies utilized in this Mendelian randomization study.

**Exposure**	**Data source (PMID)**	**Sample size**	**%European**	**Covariates adjusted in research**
Homocysteine (SD of log transformed)	PMID: 23824729	44,147	100	Age and sex, and principal components in individual studies where applicable
Vitamin B12 (SD of quantile transformed)	PMID: 23754956	45,576	100	Age, year of birth, sex, and the first principal component
Folate (SD of quantile transformed)		37,465		
Vitamin B6 (SD)	PMID: 19303062	1864	100	Not reported
**Outcomes**	**Data source (PMID)**	**Sample size** **(% cases)**	**%European**	**Covariates adjusted in GWAS**
Body fat percentage (SD)	The UK Biobank study (UKB, 25826379)	331,117	100	Age, sex, and ten principal genetic components
Trunk fat percentage (SD)				
Left arm fat percentage (SD)				
Right arm fat percentage (SD)				
Left leg fat percentage (SD)				
Right leg fat percentage (SD)				
Pediatric bone mineral density (normalization)	PMID: 28743860	8327	100	Age, sex, height, fat percent, principal components, and measurement center when applicable
Lean body mass (normalization)				
Whole body lean mass (SD)	The GEFOS Consortium (28724990)	38,292	100	Age, age^2^, sex, height, fat mass, principal components, and measurement center when applicable
Appendicular lean mass (SD)		28,330	100	
Knee osteoarthritis	The UK Biobank study (UKB, 30664745, 29559693)	455,221 (16.9%)	100	Age, sex, and ten principal genetic components
Hip osteoarthritis				
Hospital-diagnosed osteoarthritis		327,918 (9.4%)		
Musculoskeletal system diseases	The FinnGen Consortium	218,792 (52.9%)	100	Age, sex, the first ten genetic principal components, and genotyping batch
Soft tissue disorders		218,792 (23.4%)		
Osteoporosis with pathological fracture		173,619 (0.5%)		
**Confounders**	**Data source (PMID)**	**Sample size**	**%European**	**Covariates adjusted in research**
Years education attained (SD)	SSGAC (30038396)	766,345	100	Sex, birth year, their interaction, and 10 principal components of the genetic relatedness matrix
Smoking Heaviness (SD of cigarettes per day)	GSCAN (30643251)	337,334	100	Age, sex, age × sex interaction, and the first ten genetic principle components
Alcohol (SD of log-transformed drinks per week)		941,280		

GEFOS, GEnetic Factors for OSteoporosis Consortium; GSCAN, GWAS and Sequencing Consortium of Alcohol and Nicotine use; SSGAC, Social Science Genetic Association Consortium. The summary statistics data of UKB can be downloaded from Nealelab (http://www.nealelab.is/uk-biobank). The summary statistics data in FinnGen Consortium can be downloaded from Google cloud storage free of charge (https://www.finngen.fi/en/access_results).

### Investigating possible sources of horizontal pleiotropy

We also evaluated the possible association between the genetic instruments and sources of horizontal pleiotropy that could contribute to the violation of the Instrument Strength is Independent of Direct Effect (InSIDE) assumption, which means some risk factors predicted by the instruments are the potential causes of both the exposures and outcomes. These included the degree of education (SSGAC) (48) and the smoking and alcohol use rate (GSCAN) (49). The details of these variables are also presented in Table 1.

### Exposures

As the exposures increased, B vitamins were measured at the serum level, and Hcy was measured at the blood level. Both B vitamins and Hcy were transformed into one standard deviation (SD) unit.

### Outcomes

The dichotomous outcomes were attributable to musculoskeletal system diseases, soft tissue disorders, osteoporosis with pathological fractures, knee osteoarthritis, hip osteoarthritis, and hospital-diagnosed osteoarthritis. The continued outcomes were lean mass and fat percentage in various body regions (normally transformed or SD transformed).

### Harmonizing allele

The genetic information from the exposure GWAS and the outcome GWAS was merged based on the alignment of the effect alleles and the corresponding effect directions. We also employed the effect of allele frequency to ensure that palindromic genetic instruments are properly harmonized. For SNPs not available in outcome datasets, we employed proxy SNPs with r^2^ > 0.8 for the specific B vitamins or Hcy-associated SNPs. Absent SNPs without befitting proxies for outcome in the GWAS summary statistics were excluded from the subsequent analyses.

### Statistical analysis

**Figure 3:**
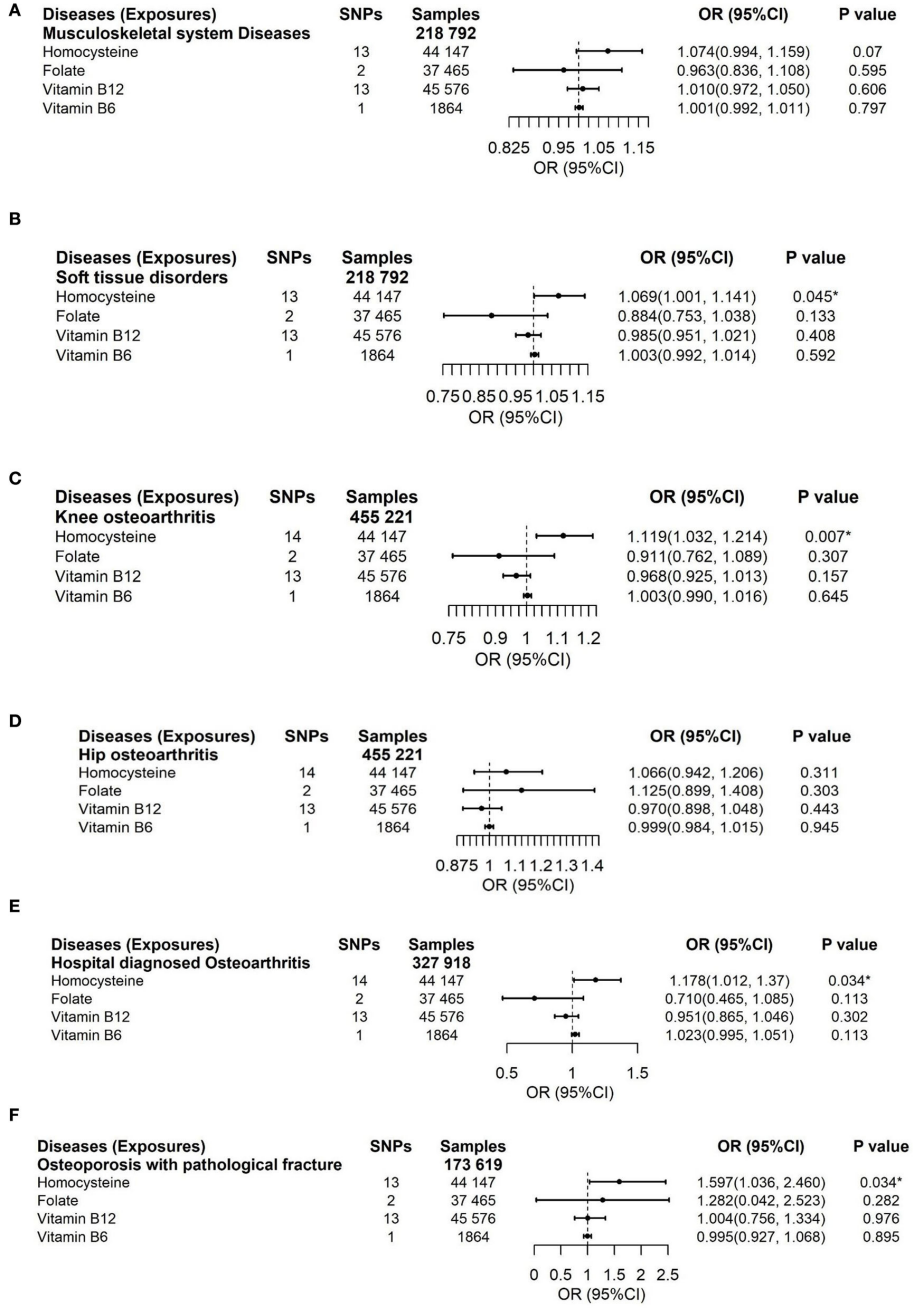
Effects of genetically predicted circulating homocysteine, vitamin B6, folate, and vitamin B12 on musculoskeletal system diseases **(A)**, soft tissue disorders **(B)**, knee osteoarthritis **(C)**, hip osteoarthritis **(D)**, hospital-diagnosed osteoarthritis **(E)**, and osteoporosis with pathological fracture **(F)** *via* IVW method. CI, confidence interval. The summary statistics data of UKB can be downloaded from Nealelab (http://www.nealelab.is/uk-biobank). The summary statistics data in FinnGen Consortium can be downloaded from Google cloud storage free of charge (https://www.finngen.fi/en/access_results). *P value reached the significant level; SNP, single nucleotide polymorphism.

**Figure 4:**
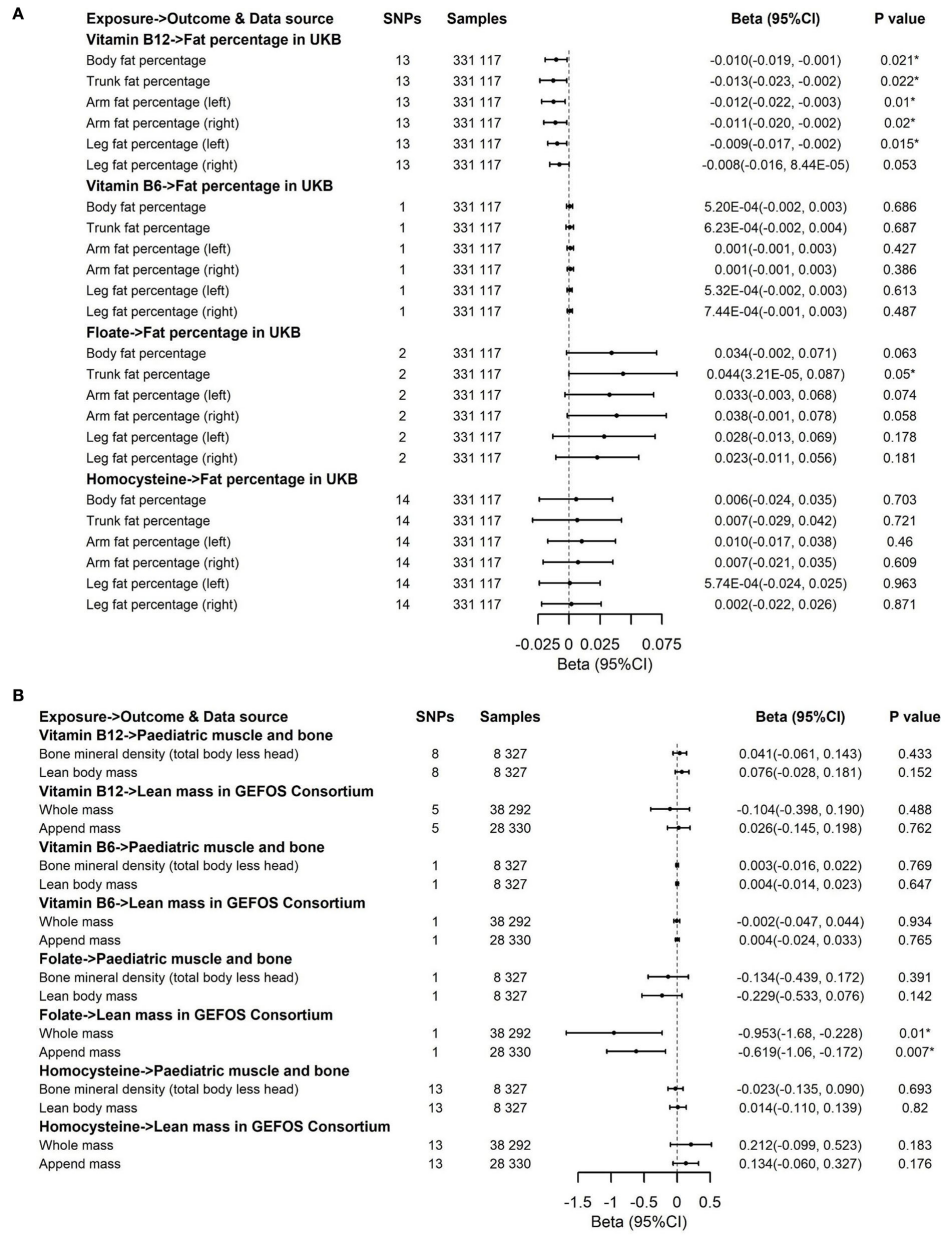
Effects of genetically predicted circulating homocysteine, vitamin B6, folate, and vitamin B12 on fat percentage **(A)** and lean mass **(B)** *via* IVW method. CI, confidence interval; GEFOS, GEnetic Factors for OSteoporosis Consortium. The summary statistics data of UKB can be downloaded from Nealelab (http://www.nealelab.is/uk-biobank). The summary statistics data in FinnGen Consortium can be downloaded from Google cloud storage free of charge (https://www.finngen.fi/en/access_results). ^*^P value reached the significant level; SNP, single nucleotide polymorphism.

**Figure 5:**
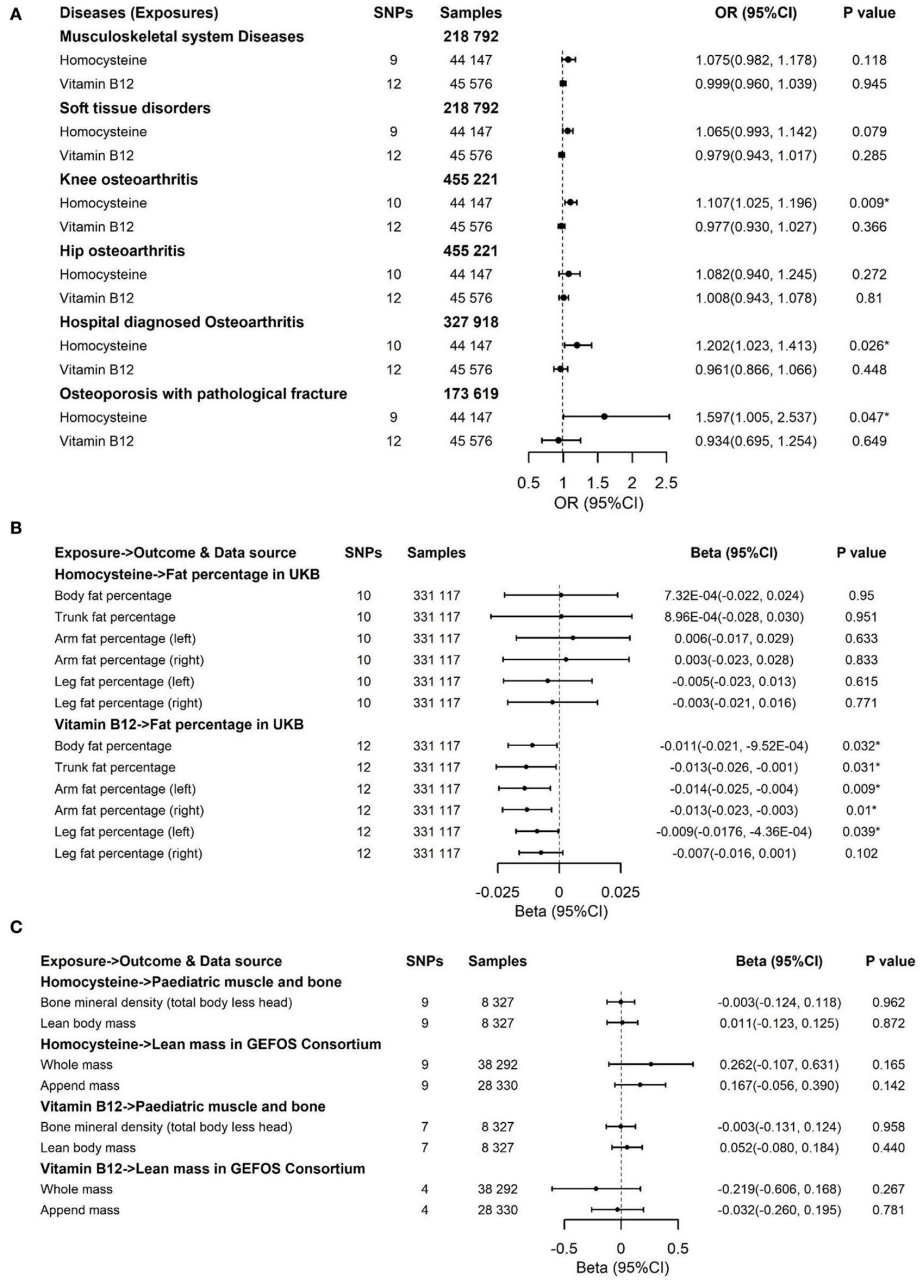
Effects of genetically predicted circulating homocysteine and vitamin B12 on musculoskeletal system diseases, soft tissue disorders, knee osteoarthritis, hip osteoarthritis, hospital-diagnosed osteoarthritis, and osteoporosis with pathological fracture **(A)**, fat percentage **(B)** and lean mass **(C)** *via* IVW method in the sensitivity analysis with exclusion of 4 pleiotropic SNPs for homocysteine and one pleotropic SNP for vitamin B12. CI, confidence interval; GEFOS, GEnetic Factors for OSteoporosis Consortium; The summary statistics data of UKB can be downloaded from Nealelab (http://www.nealelab.is/uk-biobank); The summary statistics data in FinnGen Consortium can be downloaded from Google cloud storage free of charge (https://www.finngen.fi/en/access_results). *P value reached the significant level; SNP, single nucleotide polymorphism; IVW, inverse variance weighted method.

We applied inverse variance weighting (IVW) with multiplicative random effects (50) as the main analysis (Figures 3, 4). In addition, we performed three sensitivity approaches, comprising a weighted median estimate (51), the MR-Egger (52), and the MR-PRESSO (53) method for vitamin B12 and Hcy (Supplementary Tables 5–7). The IVW stands for the weighted regression slope of the effect of SNP outcome on SNP exposure, assuming the intercept is restricted to zero (50). The weighted median allows us to provide an unbiased estimate when 50% of SNPs are invalid instruments (51). The MR-Egger can assess the horizontal pleiotropy by virtue of the *P* value for its intercept and assess after adjusting for pleiotropic effects under the InSIDE assumption, but it may get wider confidence intervals (CIs) with less statistical power (52). Using the InSIDE assumption, MR-PRESSO offers not only another statistical method for testing biases because of pleiotropy (the global test) but as well as provides a corrected estimate by means of outlier removal but also a distortion test to detect whether the results with or without outliers get similar estimates (53). By finding phenotypes correlated with the employed SNPs for vitamin B12 and Hcy in a website database (PhenoScanner V2, Supplementary Table 3, searching in April 2022) (54), a total of 1 SNP (rs602662) for vitamin B12 and 4 SNPs (rs1047891, rs548987, rs2251468, and rs838133) for Hcy correlated with blood lipids or other phenotypes tended to exert pleiotropic effects (Supplementary Table 3) (55). An additional sensitivity analysis that excluded these five SNPs was conducted to ensure the robustness of the main analysis (Figure 5). We also performed Cochran's Q statistic to explore the degree of heterogeneity (56) among the SNPs in each analysis (Supplementary Tables 5, 6). Power was calculated using an online approach (Supplementary Table 4) instead of providing supporting evidence of the strength of genetic instruments like *F*-statistics (57). The odds ratios (ORs) for dichotomous outcomes and betas for continuous outcomes with their corresponding 95% CI were scaled to one SD elevation in genetically predicted B vitamins and Hcy.

**Figure 6:**
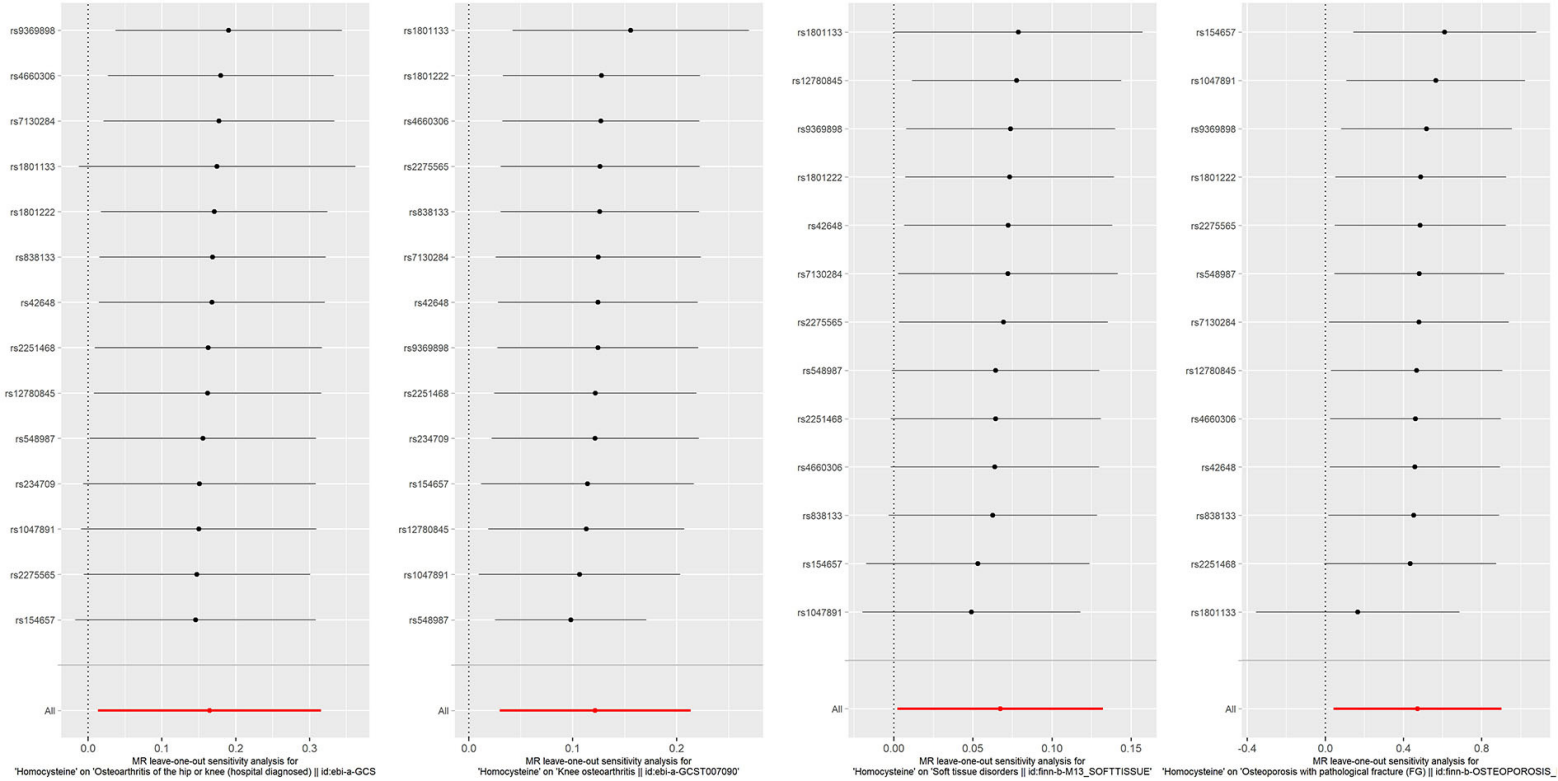
Forest plot for leave-one-out analysis of the significant IVW results of homocysteine with soft tissue disorders, knee osteoarthritis, hospital-diagnosed osteoarthritis, and osteoporosis with pathological fracture, with each point denoting the causal effect by IVW after removing the specific SNP on the left side. IVW, inverse variance weighted method.

**Figure 7:**
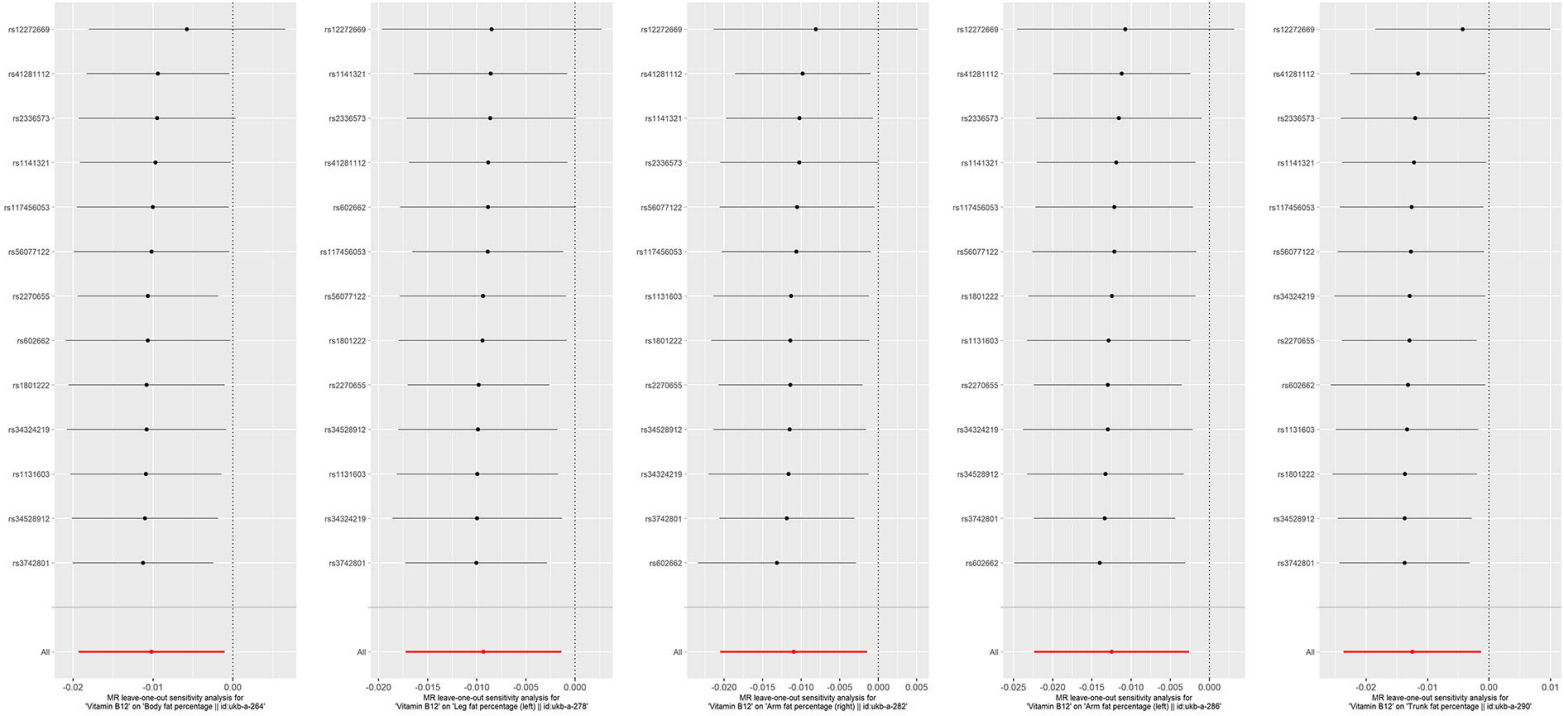
Forest plot for leave-one-out analysis of the significant IVW results of vitamin B12 with fat percentage, with each point denoting the causal effect by IVW after removing the specific SNP on the left side. IVW, inverse variance weighted method.

Because overlapping samples between two datasets would bias the evaluated causal effect, we carried out linkage disequilibrium score regression (LDSC) to estimate sample overlap by using the LD Hub (http://ldsc.broadinstitute.org/) (58). For the significant results that Hcy or B vitamins affect fat or musculoskeletal phenotypes, reverse causation analyses were conducted to rule out the possibility that fat and musculoskeletal phenotypes causally affected Hcy or B vitamins by applying fat and musculoskeletal phenotypes' associated SNPs as IVs (Supplementary Table 10). Moreover, multivariable MR was performed to account for the possibility that genetic instruments may have violated the InSIDE assumption *via* conditioning on the possible confounders. This method admits the direct effects of multiple variables on an outcome to be jointly decided. The effects of Hcy and B vitamins on fat and musculoskeletal phenotypes were evaluated in analyses that adjusted for potential confounders, including educational attainments, smoking, and alcohol usage (Supplementary Tables 8, 9). We also employed a leave-one-out analysis to ensure the robustness of the significant main findings (Figures 6, 7).

All analyses were conducted *via* R Version 4.1.0 using the R packages (“TwoSampleMR”) (59), “MRPRESSO” (53), and “MendelianRandomization” (60). Estimates with *P* < 0.05 in both the IVW and MR-PRESSO models or significant results after Bonferroni correction (0.05/*N*, where *N* is 4 exposures × 16 outcomes in this study) were considered as robust associations to control the type I error under multiple testing; estimates with *P* values above the corrected significance threshold but < 0.05 in the IVW or MR-PRESSO model and the same direction through all analyses were deemed as suggestive associations (61, 62).

## Results

To deal with the concern over bias in the evaluated causal effect because of overlapping samples in the exposure GWAS and outcome GWAS, LDSC was employed to acquire approximately zero intercepts of genetic covariance <10^−3^ in pairs of exposure-outcome GWAS (*P* > 0.1 by z-test in all pairs, data not shown), suggesting approximately no sample overlap in pairs of two datasets. For the dichotomous outcomes, including musculoskeletal system diseases, soft tissue disorders, osteoporosis with pathological fracture, knee osteoarthritis, hip osteoarthritis, and hospital-diagnosed osteoarthritis, we noticed that there was a significant association between increased genetically predicted Hcy levels and a higher risk of soft tissue disorders, osteoporosis with pathological fracture, knee osteoarthritis, and hospital-diagnosed osteoarthritis (Figure 3). For 1-SD elevations in genetically predicted Hcy concentrations, the ORs were 1.069 (95% CI, 1.001, 1.141; *P* = 0.045) for soft tissue disorders, 1.119 (95% CI, 1.032, 1.214; *P* = 0.007) for knee osteoarthritis, 1.178 (95% CI, 1.012, 1.37; *P* = 0.034) for hospital-diagnosed osteoarthritis, and 1.597 (95% CI, 1.036, 2.46; *P* = 0.034) for osteoporosis with pathological fractures. Results were kept directionally consistent in the weighted median model, MR-Egger, and MR-PRESSO approaches (Supplementary Tables 5, 7). We observed no heterogeneity based on Cochran's Q statistic and no pleiotropy based on MR-Egger and MR-PRESSO approaches for B vitamins and Hcy in the analysis of these outcomes (Supplementary Tables 5–7). Multivariable Mendelian randomization, leave-one-out analysis, and reverse Mendelian randomization analysis also confirmed these significant results (Supplementary Tables 8, 10; Figure 6). Therefore, the main results of the analyses of various MR approaches conducted by IVW were relatively robust. Genetically predicted B vitamin levels were not associated with musculoskeletally relevant diseases (Figure 3). In the sensitivity analyses with the exclusion of 1 SNP for vitamin B12 and 4 SNPs for Hcy, the associations remained consistent (Figure 5A), except for a little change in the magnitude of the effect. For 1-SD elevations in genetically predicted Hcy concentrations, the ORs were 1.065 (95% CI, 0.993, 1.142; *P* = 0.079) for soft tissue disorders, 1.107 (95% CI, 1.025, 1.196; *P* = 0.009) for knee osteoarthritis, 1.202 (95% CI, 1.023, 1.413; *P* = 0.026) for hospital-diagnosed osteoarthritis, and 1.597 (95% CI, 1.005, 2.537; *P* = 0.047) for osteoporosis with pathological fracture, which was similar with the ones before excluding the four SNPs except for soft tissue disorders. Therefore, the associations of Hcy with musculoskeletally relevant diseases were relatively robust according to all those sensitivity analyses. Overall, the positive results of enhanced Hcy levels resulting in an increased risk of musculoskeletal-relevant diseases were believable for this study.

The associations of genetically predicted Hcy and B vitamin concentrations with fat percentage and lean mass are presented in Figures 4, 5B,C. There were significant associations between increased genetically predicted vitamin B12 levels and lower body fat percentage (Beta, −0.01; 95% CI, −0.019, −0.001; *P* = 0.021) and its subtypes (Trunk fat percentage: Beta, −0.013; 95% CI, −0.023, −0.002; *P* = 0.022; Left arm fat percentage: Beta, −0.012; 95% CI, −0.022, −0.003; *P* = 0.01; Right arm fat percentage: Beta, −0.011; 95% CI, −0.02, −0.002; *P* = 0.02; Left leg fat percentage: Beta, −0.009; 95% CI, −0.017, −0.002; *P* = 0.015; Right leg fat percentage: Beta, −0.008; 95% CI, −0.016, 8.44 × 10^−5^; *P* = 0.053) as well as between higher genetically predicted folate concentrations and decreased whole body lean mass (Beta, −0.953; 95% CI, −1.68, −0.228; *P* = 0.01) and appended lean mass (Beta, −0.619; 95% CI, −1.06, −0.172; *P* = 0.007) (Figure 4). Because too few SNPs were used as instruments for folate, the robustness of significant results about the associations of folate with whole-body lean mass and appended, lean mass needs further study. The results of associations between vitamin B12 and fat percentage remained consistent and significant in the weighted median model (MR-Egger and MR-PRESSO) (Supplementary Tables 6, 7). Multivariable Mendelian randomization, leave-one-out analysis, and reverse Mendelian randomization analysis also confirmed these significant results (Supplementary Tables 9, 10; Figure 7). In addition, no heterogeneity and no pleiotropy were observed based on Cochran's Q, MR-Egger, and MR-PRESSO analyses (Supplementary Tables 6, 7). In the sensitivity analysis, we excluded 1 SNP for vitamin B12 to test its direction and significance with fat percentage and other outcomes. As a result, vitamin B12 was also significantly associated with body fat mass and its subtypes (Figure 5B). Genetically predicted Hcy concentrations and vitamin B6 levels were not associated with fat percentage and lean mass (Figures 4, 5B,C, Supplementary Tables 5, 7).

For the confounders, including the degree of education, smoking, and alcohol use rate, we observed that the effect alleles for the B vitamins and Hcy instruments (harmonized to elevated B vitamin levels and Hcy concentrations) had no effect on educational attainment, smoking, or alcohol use (Supplementary Table 2), which indicated that the associations between exposure and outcomes were unlikely to be impacted by these confounders.

## Discussion

Hcy is an intermediate outcome in the course of the metabolism of the amino acid methionine. Some nutritional factors, including vitamins B6 and B12, and folate, play a primary role in the metabolism of Hcy. Insufficiencies of either of these vitamins can affect Hcy metabolism, leading to an elevation of Hcy concentrations (12, 63) (Figure 1). Additionally, a deficiency of methionine intake would also impair Hcy metabolism. Therefore, it is up to dietary levels to either elevate or reduce blood Hcy. To date, the literature that has estimated the association of obesity with the risk of hyperhomocysteinemia in the general population is restricted and scarce (12, 26, 28, 35), and the outcomes in different population settings are controversial (13, 33). The latest study found a negative association between general obesity, measured by body mass index, and the risk of hyperhomocysteinemia. It implicated that central obesity, defined by waist circumference, was positively associated with hyperhomocysteinemia (35). Body composition examination (i.e., fat percentage, lean body mass) could better predict chronic cardiovascular diseases than body mass index or waist circumference alone (64). Previously, we researched the harm of different types of obesity and developed some genetic statistics to detect genetic variation in complex diseases (14, 65–70). Assessing human body composition accurately should be considered to further explore the relationships between fat percentage, lean body mass, Hcy, and B vitamin concentrations.

In traditional observational studies, it is difficult to examine the causal associations between exposures and disease outcomes because of methodological limitations, undetectable confounding factors, and reverse causality (71). MR design uses genetic variants as instrumental variables, which are associated with exposures as proxies for a risk factor for outcomes. It then provides causal inferences about these exposures and outcomes (72). Due to the genetic variants of the offspring being inherited randomly from their parents, generally, confounding factors are scarcely possible to influence these instrumental variables. Thus, the studies based on MR designs are less subject to reverse causality and confounding factors (36).

In the present study, we have searched the genetic variants of exposures (SNPs-Hcy and SNPs-B vitamins) and outcomes (SNPs-fat percentage, SNPs-lean mass, and SNPs-musculoskeletally relevant diseases) from distinct large-scale GWAS data sources and performed the corresponding MR analysis to estimate the causal impacts of Hcy and B vitamins on the changes of fat percentage and lean mass and the risk for musculoskeletally relevant diseases. The results of our study suggested that the increased, genetically predicted vitamin B12 levels were associated with a reduced fat percentage and its subtypes. The higher genetically predicted folate concentrations affected the decreased and appended whole-body lean mass. Moreover, genetically predicted Hcy elevation could influence the elevated risk of soft tissue disorders, osteoporosis with pathological fracture, knee osteoarthritis, and hospital-diagnosed osteoarthritis.

Several mechanisms have been established to clarify the relationships between Hcy concentrations and the pathogenesis of obesity and musculoskeletal metabolism. A changed cysteine and Hcy have a corresponding reaction to the ingested methionine in obesity, in which case both could elevate in response to dietary methionine (73). This may help explain why human obesity consistently impacts the increased concentrations of Hcy and cysteine, as already explored in the existing studies (74–76). In addition, studies stated that Hcy inhibits lipolysis in adipocytes by activating the AMPK pathway (74, 77), and total plasma Hcy and cysteine can have pro-oxidant effects by promoting reactive oxygen species and concomitant endothelial dysfunction in combination with improving low-density lipoprotein oxidation, thus favoring atherogenesis and osteoarthritis (76, 77). As we know, lean mass is a vital indicator of longevity in older adults (78), and under its plasticity and regular remodeling, when the circumstances of the elevation of intramuscular lipid deposits along with obesity and insulin resistance arise, the alterations of structure and the altered capacity for glucose homeostasis would impair muscle integrity, causing persistent atrophy and lipid accumulation in lean mass, which are also risk factors for osteoporosis (3). Furthermore, Hcy could lead to impaired muscle function using the following processes: (i) decreased oxidative defense and production of the elevated reactive oxygen species, (ii) suppression of nitric oxide signaling, (iii) inflammation and its related changes, and (iv) increased stress on the endoplasmic reticulum (79). Previously, we conducted a meta-analysis implementing MR design, confirming that genetically predicted Hcy is positively associated with obesity (14), where we stated that Hcy concentrations substantially affect the process of adjusting the relationship of DNA methylation with amino acid residues upon histones. Taken together, the above evidence indicates a pathogenic role for Hcy in musculoskeletal-relevant diseases.

It has been found that folate and vitamin B12 could affect the serum concentrations of Hcy and that co-supplementation of folate and vitamin B12 was capable of reducing serum Hcy concentrations (24). Several clinical studies have explored the effects of decreasing Hcy interventions *via* co-supplementation of folate and vitamin B12 on bone metabolism. One scholar conducted a double-blind, randomized controlled study to assess the association of the combined treatment of applying folate and vitamin B12 with the incidence of stroke and osteoporotic fractures in Japanese. The findings suggested that the individuals who had obtained co-supplementation of folate and vitamin B12 not only appeared to have a significant decrease in plasma Hcy concentrations but also had efficiency in reducing the risk of osteoporotic fractures (25). Moreover, the Hcy levels of osteoporosis patients treated with a novel medication aimed at treating osteoporosis for 1 year revealed significant reductions over time compared to the basal Hcy concentrations (27). Additionally, pre-clinical studies have reported a mechanistic physiological relationship whereby vitamin B12 deficiency results in elevated adipogenesis and cholesterol biosynthesis in human cells and rats, respectively (31, 80). The increase of genetically predicted vitamin B12 was associated with this study's reduction in fat percentage, supporting the hypothesis that there is a putative correlation between lower B12 concentrations and obesity.

**Figure 8:**
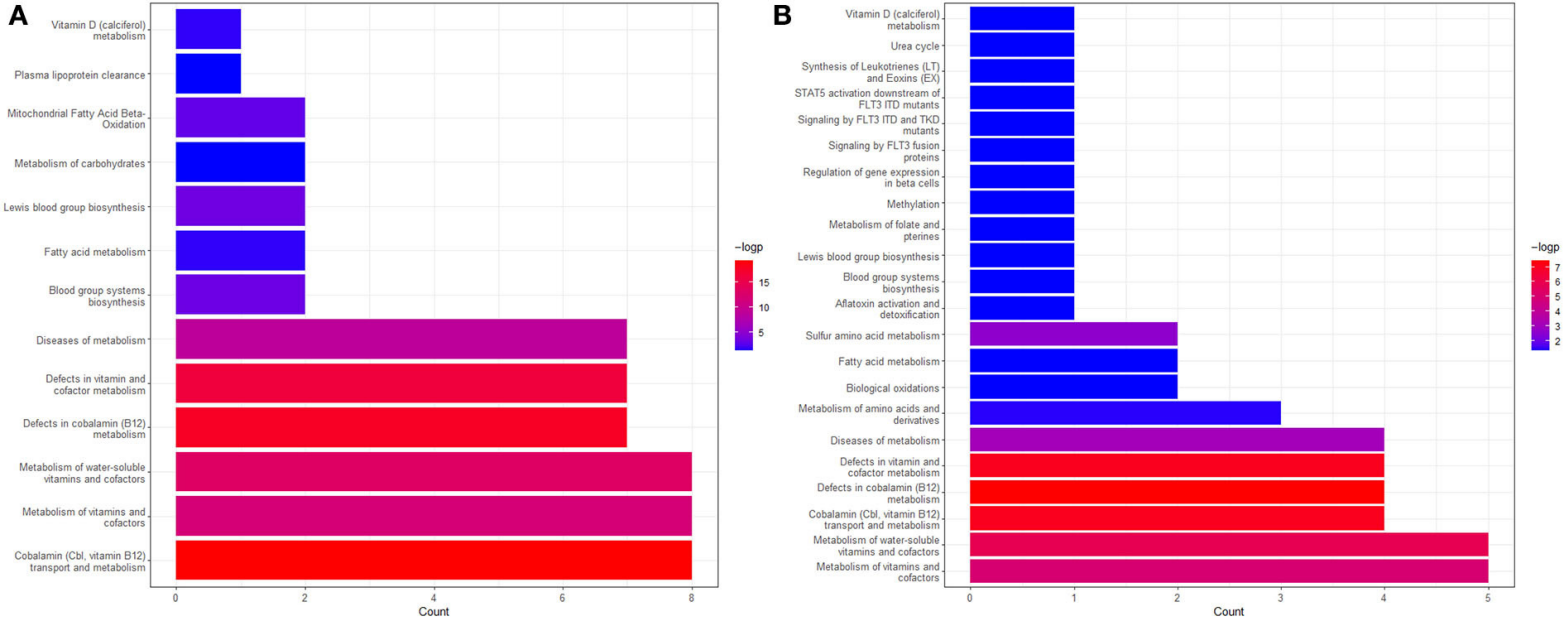
The Reactome enrichment analyses genes associated with vitamin B12 **(A)** and Hcy **(B)**. The pathways with adjusted *P* < 0.05 are left in the figure. More information can be found on the website (https://reactome.org/).

To further validate the results in this study and find more explanations for possible pathways, we conducted Reactome enrichment analyses on the genes associated with vitamin B12 and Hcy, respectively, to validate the role vitamin B12 and Hcy might play in the pathogenesis and development of fat obesity and musculoskeletally relevant diseases. Figures 8A,B depict the results of enrichment analyses of the vitamin B12 and Hcy gene sets, respectively. From Figure 8A, we observed that the genes associated with vitamin B12 were enriched in mitochondrial fatty acid beta-oxidation, which was active in the liver. A previous study revealed that inhibiting hepatic fatty acid oxidation (FAO) resulted in a systemic hormetic response that protected mice from obesity and glucose intolerance induced by a high-fat diet, which implied vitamin B12 metabolism might relate to FAO and thereby have an association with obesity (81). More importantly, a study in adult Wistar rats fed a vitamin B12-restricted diet during the maternal or postnatal period predicted higher visceral adiposity and resulted in an alteration in the metabolism of lipids in the offspring. Vitamin-restricted offspring also had higher activities of hepatic fatty acid synthase and acetyl-CoA-carboxylase and higher plasma cortisol levels (82, 83). Another study found that restriction of maternal B12, folate, and methionine at the conception stage in sheep models showed increased resistance to insulin and elevated blood pressure in their offspring (83, 84). The aforementioned findings suggest that vitamin B12 may influence body fat mainly through FAO and insulin-related pathways. In Figure 8B, except that Hcy-related genes were enriched in vitamin metabolism for vitamin B6 and B12 and were involved in the metabolism of Hcy, these genes were also enriched in the vitamin D metabolic pathway, which played a crucial role in the musculoskeletal systems (85, 86). The potential pathways by which Hcy and vitamin B12 are involved in the fat, and musculoskeletal systems require further investigation.

The strengths of this study lie in the application of Mendelian randomization, which could diminish residual confounding and reduce the likelihood of false negatives in large GWAS. To our knowledge, this is the first study employing the MR approach to evaluate the causal associations of genetically predicted Hcy and B vitamin concentrations with fat and musculoskeletally relevant phenotypes *via* several independent data sources. Additionally, combining various non-overlapping data sources and employing more SNPs associated with Hcy and B vitamins could guarantee the sample size, explain the phenotypic variance, respectively, and ensure statistical power, even though certain weak associations have been overlooked. The concerns about vitamin B12 and Hcy may not be independent variables for interfering with causality. Actually, no matter whether it is vitamin B12 or Hcy, the three conditions (Figure 2) for conducting an MR study are satisfied. To avoid horizontal pleiotropy because of the interlink between vitamin B12 and Hcy, we used strict screening criteria for selecting SNPs as instrumental variables. In other words, these instruments could infer the causal effects of vitamin B12 on fat and musculoskeletal diseases and Hcy on fat and musculoskeletal diseases, respectively. We confined the population in this study to subjects of European ancestry to diminish population structure bias, which impeded the generalization of our results to other populations. Another limitation is the possibility of pleiotropy. Nonetheless, our sensitivity analyses produced directionally consistent findings with the same significance in different sensitivity approaches (weighted median method, MR-Egger, MR-PRESSO, reverse Mendelian randomization). We also tested the confounding factors, including education, smoking, and alcohol use, which might confuse the associations between exposures and outcomes. As a result, these confounders cannot confuse the associations (multivariable MR). Moreover, the results of MR-Egger and MR-PRESSO showed no pleiotropic effects in most analyses, so pleiotropy and confounding did not bias our results. Furthermore, several Hcy-associated SNPs or vitamin B12-associated SNPs affecting the genetic predisposition to blood pressure or lipid traits were excluded. The magnitude of our findings did not change significantly, confirming our results' robustness. Moreover, dietary intake was not considered, and the variation in each of the exposures that SNPs explained was small. Unhealthy lifestyles were also major factors that need further consideration. Moreover, 1 SNP for vitamin B6 and 2 SNPs for folate may provide insufficient power to infer the associations. As the involved population encompasses distinct demographic or clinical characteristics and the complex relationship between B vitamins and Hcy is described, more detailed and comprehensive studies about causal effects are urgently needed to further confirm the associations and degrees of Hcy and B vitamins with the risk of fat and musculoskeletally relevant diseases.

## Conclusions

In summary, the present study indicated causalities between genetically predicted Hcy increase and the elevated risk of soft tissue disorders, osteoporosis with pathological fracture, knee osteoarthritis, and hospital-diagnosed osteoarthritis, as well as genetically predicted vitamin B12 enhancement and the decrease of fat percentage and its subtypes, revealing that disordered expressions of Hcy and vitamin B12 might play a role in the pathogenesis and development of fat obesity and musculoskeletally relevant diseases, respectively. The supplementation of vitamin B12 and lowering Hcy therapy could be potential options for preventing and treating obesity and musculoskeletal-related diseases. As for folate, further evidence is needed to confirm its relationship with lean mass because of the limited instruments for folate employed in this study.

## Data availability statement

Publicly available datasets were analyzed in this study. This data can be found here: The authors thank the UK Biobank study, and the summary statistics data of UKB can be download from Nealelab (http://www.nealelab.is/uk-biobank). The authors thank the FinnGen consortium, and the summary statistics data in FinnGen Consortium can be downloaded from Google cloud storage free of charge (https://www.finngen.fi/en/access_results). The authors thank SSGAC for supplying the summary statistics of years of education attainment, which have been downloaded from https://www.thessgac.org/home. The authors thank GSCAN for supplying the summary statistics of smoking and alcohol use phenotypes, which have been downloaded from https://conservancy.umn.edu/handle/11299/201564.

## Author contributions

Study concept and design and drafting of the manuscript: LF and Y-QH. Acquisition of data and critical revision of the manuscript for important intellectual content: LF, Y-QH, and YW. Analysis and interpretation of data: LF. All authors have read and approved the final version of the manuscript.

## Funding

This study was supported by grants to LF and Y-QH from the National Natural Science Foundation of China (Grants No. 82204063, 11971117, and 11571082).

## Conflict of interest

The authors declare that the research was conducted in the absence of any commercial or financial relationships that could be construed as a potential conflict of interest.

## Publisher's note

All claims expressed in this article are solely those of the authors and do not necessarily represent those of their affiliated organizations, or those of the publisher, the editors and the reviewers. Any product that may be evaluated in this article, or claim that may be made by its manufacturer, is not guaranteed or endorsed by the publisher.
