# Supplementary material for: Causal effects of B vitamins and homocysteine on obesity and musculoskeletal diseases: A Mendelian randomization study
Source: Front Nutr. 2022 Nov 24;9:1048122. doi: 10.3389/fnut.2022.1048122 (PMC9731309; doi:10.3389/fnut.2022.1048122)
Supplement: Supplementary file 1 [file Data_Sheet_1.docx]

**Supplementary materials for**

**Causal effects of B vitamins, homocysteine on fat and musculoskeletal diseases: A Mendelian randomization study**

Liwan Fu, Yuquan Wang, Yue-Qing Hu

**Supplementary Table 1.** Descriptions of instrumental variables

**Supplementary Table 2.** Genetic association of B vitamins, homocysteine instruments with confounders

**Supplementary Table 3.** Pleiotropic examinations with applied SNPs for B vitamins and homocysteine

**Supplementary Table 4.** Power evaluation

**Supplementary Table 5.** Associations of genetically predicted circulating homocysteine with fat and musculoskeletal phenotypes in sensitivity analyses via Heterogeneity test, Weighted median and MR-Egger

**Supplementary Table 6.** Associations of genetically predicted circulating vitamin B12 with fat and musculoskeletal phenotypes in sensitivity analyses via Heterogeneity test, Weighted median and MR-Egger

**Supplementary Table 7.** Associations of genetically predicted circulating homocysteine and vitamin B12 with fat and musculoskeletal phenotypes in the MR-PRESSO analysis

**Supplementary Table 8.** Validating the significant IVW results of homocysteine with soft tissue disorders, knee osteoarthritis, hospital diagnosed osteoarthritis, and osteoporosis with pathological fracture by using the Multivariable Mendelian randomization adjusting education attained, smoking and alcohol usage

**Supplementary Table 9.** Validating the significant IVW results of vitamin B12 with fat percentage by using the Multivariable Mendelian randomization adjusting education attained, smoking and alcohol usage

**Supplementary Table 10.** The significant IVW results of vitamin B12 with fat percentage, homocysteine with soft tissue disorders, knee osteoarthritis, hospital diagnosed osteoarthritis, and osteoporosis with pathological fracture are validated for the possible reverse associations via the reverse Mendelian randomization analysis

**Supplementary Table 1.** Descriptions of instrumental variables.

| SNP | Chr | Position | EA | OA | EAF | Effect | SE | *P* value | Mapped Gene | Exposure |
| --- | --- | --- | --- | --- | --- | --- | --- | --- | --- | --- |
| rs4654748 | 1 | 21786068 | T | C | 0.50 | 1.450 | 0.280 | 8.30E-18 | *ALPL* | Vitamin B6 |
| rs2270655 | 4 | 146576418 | G | C | 0.94 | 0.066 | 0.018 | 2.20E-13 | *MMAA* | Vitamin B12 |
| rs1141321 | 6 | 49412433 | C | T | 0.63 | 0.061 | 0.007 | 3.60E-26 | *MUT* | Vitamin B12 |
| rs7788053 | 7 | 86773722 | A | G | 0.25 | 0.046 | 0.007 | 1.70E-10 | *FUT6* | Vitamin B12 |
| rs1801222 | 10 | 17156151 | G | A | 0.59 | 0.110 | 0.007 | 3.30E-75 | *CUBN* | Vitamin B12 |
| rs56077122 | 10 | 17207015 | A | C | 0.34 | 0.087 | 0.009 | 4.80E-21 | *CUBN/TRDMT1* | Vitamin B12 |
| rs12272669 | 11 | 71392610 | A | G | 0.01 | 0.510 | 0.007 | 3.00E-09 | *MMACHC* | Vitamin B12 |
| rs34324219 | 11 | 59623378 | C | A | 0.88 | 0.210 | 0.007 | 1.10E-111 | *TCN1* | Vitamin B12 |
| rs34528912 | 11 | 59631535 | T | C | 0.04 | 0.170 | 0.021 | 2.10E-15 | *TCN1* | Vitamin B12 |
| rs117456053 | 11 | 59616831 | G | A | 0.98 | 0.160 | 0.026 | 1.90E-09 | *TCN1* | Vitamin B12 |
| rs41281112 | 13 | 100518634 | C | T | 0.95 | 0.170 | 0.020 | 8.90E-35 | *CLYBL* | Vitamin B12 |
| rs3742801 | 14 | 74759006 | T | C | 0.29 | 0.045 | 0.009 | 1.70E-13 | *ABCD4* | Vitamin B12 |
| rs2336573 | 19 | 8367709 | T | C | 0.03 | 0.320 | 0.007 | 8.40E-59 | *CD320* | Vitamin B12 |
| rs602662 | 19 | 49206985 | A | G | 0.60 | 0.160 | 0.007 | 2.40E-139 | *FUT2* | Vitamin B12 |
| rs1131603 | 22 | 31018975 | C | T | 0.06 | 0.190 | 0.017 | 4.90E-49 | *TCN2* | Vitamin B12 |
| rs1801133 | 1 | 11856378 | G | A | 0.67 | 0.096 | 0.008 | 9.50E-53 | *MTHFR* | Folate |
| rs652197 | 11 | 71849741 | C | T | 0.18 | 0.069 | 0.011 | 1.40E-12 | *FOLR3* | Folate |
| rs1801133 | 1 | 11856378 | A | G | 0.34 | 0.158 | 0.007 | 4.30E-104 | *MTHFR* | Homocysteine |
| rs2275565 | 1 | 237048676 | G | T | 0.79 | 0.054 | 0.009 | 2.00E-10 | *MTR* | Homocysteine |
| rs4660306 | 1 | 45978675 | T | C | 0.33 | 0.043 | 0.007 | 2.30E-09 | *MMACHC* | Homocysteine |
| rs1047891 | 2 | 211540507 | A | C | 0.33 | 0.086 | 0.008 | 4.60E-27 | *CPS1* | Homocysteine |
| rs9369898 | 6 | 49382193 | A | G | 0.62 | 0.045 | 0.007 | 2.20E-10 | *MUT* | Homocysteine |
| rs548987 | 6 | 25869371 | C | G | 0.13 | 0.060 | 0.010 | 1.10E-08 | *SLC17A3* | Homocysteine |
| rs42648 | 7 | 89977760 | G | A | 0.60 | 0.039 | 0.007 | 2.00E-08 | *GTPB10* | Homocysteine |
| rs1801222 | 10 | 17156151 | A | G | 0.34 | 0.045 | 0.007 | 8.40E-10 | *CUBN* | Homocysteine |
| rs12780845 | 10 | 17223244 | A | G | 0.65 | 0.053 | 0.009 | 7.80E-10 | *CUBN* | Homocysteine |
| rs7130284 | 11 | 89148372 | C | T | 0.93 | 0.124 | 0.013 | 1.90E-20 | *NOX4* | Homocysteine |
| rs2251468 | 12 | 121405126 | C | A | 0.35 | 0.051 | 0.007 | 1.30E-12 | *HNF1A* | Homocysteine |
| rs154657 | 16 | 89708096 | A | G | 0.47 | 0.096 | 0.007 | 1.70E-43 | *DPEP1* | Homocysteine |
| rs838133 | 19 | 49259529 | A | G | 0.45 | 0.042 | 0.007 | 7.50E-09 | *FUT2* | Homocysteine |
| rs234709 | 21 | 44486964 | C | T | 0.55 | 0.072 | 0.007 | 3.90E-24 | *CBS* | Homocysteine |

Chr, chromosome; EA, effect allele; EAF, effect allele frequency; OA, other allele; SE, standard error; SNP, single nucleotide polymorphism.

**Supplementary Table 2.** Genetic association of B vitamins, homocysteine instruments with confounders.

| Exposure | Confounders | Effect | SE | *P* value |
| --- | --- | --- | --- | --- |
| Vitamin B6 | Education (SD) | -1.10E-04 | 2.83E-04 | 0.685 |
|  | Alcohol (SD of log transformed drinks per week) | -9.60E-04 | 1.35E-03 | 0.476 |
|  | Tobacco (SD of cigarettes per week) | 5.82E-03 | 3.98E-03 | 0.144 |
| Vitamin B12 | Education (SD) | 1.40E-03 | 1.44E-03 | 0.332 |
|  | Alcohol (SD of log transformed drinks per week) | 0.013 | 9.92E-03 | 0.183 |
|  | Tobacco (SD of cigarettes per week) | -0.018 | 0.013 | 0.177 |
| Folate | Education (SD) | 6.73E-03 | 0.004 | 0.095 |
|  | Alcohol (SD of log transformed drinks per week) | -0.025 | 0.019 | 0.196 |
|  | Tobacco (SD of cigarettes per week) | 0.061 | 0.054 | 0.255 |
| Homocysteine | Education (SD) | -1.94E-03 | 2.21E-03 | 0.382 |
|  | Alcohol (SD of log transformed drinks per week) | 7.89E-03 | 0.012 | 0.499 |
|  | Tobacco (SD of cigarettes per week) | 0.062 | 0.039 | 0.112 |

Effects were obtained from the random-effects invariance weighted median model; SE, standard error.

**Supplementary Table 3.** Pleiotropic examinations by applying SNPs for B vitamins and homocysteine.

| Exposure/SNP | Mapped Gene | Effect allele | Phenotypes | Direction |
| --- | --- | --- | --- | --- |
| Vitamin B6 | | | | |
| rs4654748 | *ALPL* | T | Alkaline phosphatase | - |
| Vitamin B12 | | | | |
| rs2270655 | *MMAA* | G | NA |  |
| rs1141321 | *MUT* | C | NA |  |
| rs7788053 | *FUT6* | A | NA |  |
| rs1801222 | *CUBN* | G | NA |  |
| rs56077122 | *CUBN/TRDMT1* | A | NA |  |
| rs12272669 | *MMACHC* | A | NA |  |
| rs34324219 | *TCN1* | C | Self-reported pernicious anaemia | - |
| rs34528912 | *TCN1* | T | NA |  |
| rs117456053 | *TCN1* | G | NA |  |
| rs41281112 | *CLYBL* | C | NA |  |
| rs3742801 | *ABCD4* | T | NA |  |
| rs2336573 | *CD320* | T | NA |  |
| rs602662 | *FUT2* | A | Self-reported high cholesterol | + |
|  |  |  | Alkaline phosphatase | - |
|  |  |  | Cholelithiasis | + |
|  |  |  | Sodium in urine | - |
|  |  |  | Mean platelet volume | - |
|  |  |  | Alcohol intake frequency | - |
|  |  |  | Total cholesterol | + |
|  |  |  | Crohns disease | + |
|  |  |  | Bring up phlegm or sputum/mucus on most days | + |
|  |  |  | Treatment with simvastatin | + |
| rs1131603 | *TCN2* | C | NA |  |
| Folate | | | | |
| rs1801133 | *MTHFR* | G | Diastolic blood pressure | - |
|  |  |  | Mean corpuscular hemoglobin | - |
| rs652197 | *FOLR3* | C | NA |  |
| Homocysteine | | | | |
| rs1801133 | *MTHFR* | A | Diastolic blood pressure | - |
|  |  |  | Mean corpuscular hemoglobin | - |
| rs2275565 | *MTR* | G | NA |  |
| rs4660306 | *MMACHC* | T | NA |  |
| rs1047891 | *CPS1* | A | Whole body water mass | + |
|  |  |  | Whole body and trunk fat-free mass | + |
|  |  |  | Impedance of whole body | - |
|  |  |  | Basal metabolic rate | + |
|  |  |  | Platelet count | + \| - |
|  |  |  | Creatinine in urine | + |
|  |  |  | Chronic kidney disease | + |
|  |  |  | Weight | + |
|  |  |  | Mother's heart disease | - |
|  |  |  | Mean corpuscular hemoglobin | + |
|  |  |  | Mean corpuscular volume | + |
|  |  |  | High density lipoprotein cholesterol | + \| - |
|  |  |  | Systolic blood pressure | - |
|  |  |  | Plateletcrit | - |
|  |  |  | Eosinophil percentage of white cells | + |
|  |  |  | Fibrinogen | + |
|  |  |  | Headache | + |
|  |  |  | White blood cell count | - |
|  |  |  | Hip circumference | + |
| rs9369898 | *MUT* | A | NA |  |
| rs548987 | *SLC17A3* | C | Mean corpuscular hemoglobin | - |
|  |  |  | Red cell distribution width | + |
|  |  |  | Hemoglobin concentration | - |
|  |  |  | Primary sclerosing cholangitis | + |
|  |  |  | Mean corpuscular volume | - |
|  |  |  | Hematocrit | - |
|  |  |  | Intestinal malabsorption | + |
|  |  |  | Reticulocyte count | - |
|  |  |  | IgA deficiency | + |
|  |  |  | Schizophrenia | - |
|  |  |  | Lymphocyte count | - |
|  |  |  | Forced expiratory volume | - |
|  |  |  | Leg fat mass | + |
|  |  |  | Monocyte count | - |
|  |  |  | Self-reported gout | + |
|  |  |  | Serum urate | + |
|  |  |  | White blood cell count | - |
|  |  |  | Self-reported sarcoidosis | + |
|  |  |  | Body mass index | + |
|  |  |  | Headache | - |
| rs42648 | *GTPB10* | G | Impedance of arm right | + |
| rs1801222 | *CUBN* | A | NA |  |
| rs12780845 | *CUBN* | A | NA |  |
| rs7130284 | *NOX4* | C | NA |  |
| rs2251468 | *HNF1A* | C | Low density lipoprotein | + |
|  |  |  | Coronary artery disease | + |
|  |  |  | Plateletcrit | - |
|  |  |  | Total cholesterol | + |
|  |  |  | Gamma glutamyl transferase | - |
|  |  |  | C-reactive protein | - |
|  |  |  | Mean corpuscular hemoglobin | + |
| rs154657 | *DPEP1* | A | Self-reported hypertension | - |
|  |  |  | Mean corpuscular volume | + |
|  |  |  | Hematocrit | + |
|  |  |  | log eGFR creatinine in non-diabetics | - |
| rs838133 | *FUT2* | A | Sodium in urine | - |
|  |  |  | Mean platelet volume | - |
|  |  |  | Hip circumference | - |
|  |  |  | Sitting height | - |
|  |  |  | Percentage of total caloric intake from macronutrients protein | Not reported |
|  |  |  | Dietary macronutrient intake | - |
|  |  |  | Total cholesterol | + |
|  |  |  | Cholelithiasis | + |
| rs234709 | *CBS* | C | Blood and toenail selenium levels | Not reported |

SNPs in red color means pleiotropy; NA, not available; SNP, single nucleotide polymorphism. These associations were identified at the genome-wide significance level from the PhenoScanner V2, a database of human genotype-phenotype associations (http://www.phenoscanner.medschl.cam.ac.uk/).

**Supplementary Table 4.** Power evaluation.

| Outcomes | Source | Sample size | Beta_B6_ | Beta_B12_ | Beta_folate_ | Beta_tHcy_ | ~6% of variance  Beta at 80% power | | ~1% of variance  Beta at 80% power | |
| --- | --- | --- | --- | --- | --- | --- | --- | --- | --- | --- |
|  |  |  |  |  |  |  | ≤ lower | ≥ upper | ≤ lower | ≥ upper |
| Body fat percentage | UKB | 331,117 | 5.20E-04 | -0.010 | 0.034 | 0.006 | -0.02 | 0.02 | -0.049 | 0.049 |
| Trunk fat percentage | UKB | 331,117 | 6.24E-04 | -0.013 | 0.044 | 0.007 | -0.02 | 0.02 | -0.049 | 0.049 |
| Left arm fat percentage | UKB | 331,117 | 1.02E-03 | -0.013 | 0.033 | 0.010 | -0.02 | 0.02 | -0.049 | 0.049 |
| Right arm fat percentage | UKB | 331,117 | 1.12E-03 | -0.011 | 0.038 | 0.007 | -0.02 | 0.02 | -0.049 | 0.049 |
| Left leg fat percentage | UKB | 331,117 | 5.32E-04 | -0.010 | 0.028 | 0.001 | -0.02 | 0.02 | -0.049 | 0.049 |
| Right leg fat percentage | UKB | 331,117 | 7.44E-04 | -0.008 | 0.023 | 0.002 | -0.02 | 0.02 | -0.049 | 0.049 |
| Pediatric bone mineral density | PMID: 28743860 | 8327 | 2.82E-03 | 0.041 | -0.134 | -0.023 | -0.126 | 0.126 | -0.33 | 0.33 |
| Lean body mass | PMID: 28743860 | 8327 | 4.40E-03 | 0.076 | -0.229 | 0.0145 | -0.126 | 0.126 | -0.33 | 0.33 |
| Whole body lean mass | GEFOS | 38,292 | -1.93E-03 | -0.104 | -0.953 | 0.212 | -0.058 | 0.058 | -0.145 | 0.145 |
| Appendicular lean mass | GEFOS | 28,330 | 4.35E-03 | 0.026 | -0.619 | 0.136 | -0.068 | 0.068 | -0.17 | 0.17 |

| Outcomes | Source | Sample size | Case % | OR_B6_ | OR_B12_ | OR_folate_ | OR_tHcy_ | ~6% of variance  OR at 80% power | | ~1% of variance  OR at 80% power | |
| --- | --- | --- | --- | --- | --- | --- | --- | --- | --- | --- | --- |
|  |  |  |  |  |  |  |  | ≤ lower | ≥ upper | ≤ lower | ≥ upper |
| Knee osteoarthritis | UKB | 455,221 | 16.9% | 1.003 | 0.968 | 0.911 | 1.119 | 0.955 | 1.045 | 0.891 | 1.113 |
| Hip osteoarthritis | UKB | 455,221 | 16.9% | 0.999 | 0.970 | 1.125 | 1.066 | 0.955 | 1.045 | 0.891 | 1.113 |
| Hospital diagnosed osteoarthritis | UKB | 327,918 | 9.4% | 1.023 | 0.951 | 0.710 | 1.178 | 0.932 | 1.069 | 0.835 | 1.171 |
| Diseases of the musculoskeletal system and connective tissue | FinnGen | 218,792 | 52.9% | 1.001 | 1.010 | 0.963 | 1.074 | 0.952 | 1.050 | 0.888 | 1.128 |
| Soft tissue disorders | FinnGen | 218,792 | 23.4% | 1.003 | 0.985 | 0.884 | 1.069 | 0.943 | 1.059 | 0.864 | 1.147 |
| Osteoporosis with pathological fracture | FinnGen | 173,619 | 0.5% | 0.995 | 1.004 | 1.282 | 1.597 | 0.610 | 1.390 | 0.150 | 1.950 |

ORs and Betas were obtained from the random-effects inverse-variance weighted model; NA, not available.

Power was calculated using an online tool: http://cnsgenomics.com/shiny/mRnd/.

**Supplementary Table 5.** Associations of genetically predicted circulating homocysteine with fat and musculoskeletal phenotypes in sensitivity analyses via Heterogeneity test, Weighted median and MR-Egger.

| Source | Outcome | SNPs  used | *Q* | *P (Q)* | Weighted median | | | | MR-Egger | | | | |
| --- | --- | --- | --- | --- | --- | --- | --- | --- | --- | --- | --- | --- | --- |
|  |  |  |  |  | Effect | 95% CI | | *P* | Effect | 95% CI | | *P* | *P_intercept_* |
| UKB | Body fat percentage | 14 | 53.3 | 7.97E-07 | -0.005 | -0.029 | 0.020 | 0.715 | -0.026 | -0.102 | 0.050 | 0.504 | 0.333 |
| UKB | Trunk fat percentage | 14 | 46.1 | 1.34E-05 | -0.022 | -0.050 | 0.006 | 0.125 | -0.030 | -0.120 | 0.061 | 0.521 | 0.362 |
| UKB | Left arm fat percentage | 14 | 48.3 | 5.79E-06 | 0.016 | -0.009 | 0.041 | 0.216 | -0.023 | -0.089 | 0.043 | 0.491 | 0.242 |
| UKB | Right arm fat percentage | 14 | 54.2 | 5.47E-07 | 0.014 | -0.011 | 0.040 | 0.275 | -0.033 | -0.099 | 0.033 | 0.328 | 0.160 |
| UKB | Left leg fat percentage | 14 | 50.7 | 2.22E-06 | 0.005 | -0.015 | 0.026 | 0.624 | -0.026 | -0.089 | 0.038 | 0.432 | 0.336 |
| UKB | Right leg fat percentage | 14 | 9.64 | 0.647 | 0.006 | -0.013 | 0.026 | 0.510 | -0.019 | -0.082 | 0.044 | 0.548 | 0.416 |
| PMID: 28743860 | Pediatric bone mineral density | 13 | 14.6 | 0.259 | -0.012 | -0.165 | 0.141 | 0.877 | 0.181 | -0.062 | 0.423 | 0.144 | 0.063 |
| PMID: 28743860 | Lean body mass | 13 | 16.4 | 0.173 | 0.104 | -0.050 | 0.257 | 0.185 | 0.173 | -0.094 | 0.440 | 0.204 | 0.191 |
| GEFOS | Whole body lean mass | 13 | 17.2 | 0.141 | 0.335 | -0.035 | 0.704 | 0.076 | 0.727 | 0.109 | 1.346 | 0.021 | 0.066 |
| GEFOS | Appendicular lean mass | 13 | 53.3 | 7.97E-07 | 0.342 | 0.118 | 0.566 | 0.003 | 0.497 | 0.128 | 0.867 | 0.008 | 0.029 |
| FinnGen | Diseases of the musculoskeletal system and connective tissue | 13 | 20.5 | 0.057 | 1.057 | 0.973 | 1.148 | 0.187 | 1.121 | 0.940 | 1.337 | 0.205 | 0.610 |
| FinnGen | Soft tissue disorders | 13 | 9.83 | 0.630 | 1.069 | 0.982 | 1.163 | 0.122 | 1.109 | 0.962 | 1.277 | 0.155 | 0.578 |
| UKB | Knee osteoarthritis | 14 | 16.3 | 0.233 | 1.078 | 0.977 | 1.189 | 0.134 | 1.122 | 0.906 | 1.390 | 0.291 | 0.950 |
| UKB | Hip osteoarthritis | 14 | 23.9 | 0.032 | 1.038 | 0.903 | 1.193 | 0.603 | 1.128 | 0.842 | 1.513 | 0.419 | 0.683 |
| UKB | Hospital diagnosed osteoarthritis | 14 | 8.43 | 0.814 | 1.206 | 0.985 | 1.476 | 0.069 | 1.312 | 0.935 | 1.842 | 0.117 | 0.489 |
| FinnGen | Osteoporosis with pathological fracture | 13 | 8.47 | 0.746 | 1.673 | 0.889 | 3.149 | 0.110 | 2.110 | 0.824 | 5.404 | 0.120 | 0.520 |

CI, confidence interval; GEFOS, GEnetic Factors for OSteoporosis Consortium; The summary statistics data of UKB can be download from Nealelab (http://www.nealelab.is/uk-biobank); The summary statistics data in FinnGen Consortium can be downloaded from Google cloud storage free of charge. SNP, single nucleotide polymorphism; The Q statistic was used to present the heterogeneity among estimates for each SNPs in one analysis; For continuous outcomes, Effect means the value of Beta, and for categorical outcomes, Effect is the value of OR; The P value for the intercept in the MR-Egger regression was used present the pleiotropy (P < 0.05).

**Supplementary Table 6.** Associations of genetically predicted circulating vitamin B12 with fat and musculoskeletal phenotypes in sensitivity analyses via Heterogeneity test, Weighted median and MR-Egger.

| Source | Outcome | SNPs  used | *Q* | *P (Q)* | Weighted median | | | | MR-Egger | | | | |
| --- | --- | --- | --- | --- | --- | --- | --- | --- | --- | --- | --- | --- | --- |
|  |  |  |  |  | Effect | 95% CI | | *P* | Effect | 95% CI | | *P* | *P_intercept_* |
| UKB | Body fat percentage | 13 | 12.0 | 0.447 | -0.013 | -0.023 | -0.002 | 0.023 | -0.020 | -0.034 | -0.006 | 0.006 | 0.085 |
| UKB | Trunk fat percentage | 13 | 12.6 | 0.397 | -0.019 | -0.032 | -0.005 | 0.006 | -0.028 | -0.045 | -0.011 | 0.001 | 0.025 |
| UKB | Left arm fat percentage | 13 | 14.4 | 0.274 | -0.014 | -0.025 | -0.003 | 0.012 | -0.018 | -0.034 | -0.002 | 0.026 | 0.367 |
| UKB | Right arm fat percentage | 13 | 13.6 | 0.330 | -0.010 | -0.021 | 0.001 | 0.068 | -0.017 | -0.032 | -0.001 | 0.035 | 0.361 |
| UKB | Left leg fat percentage | 13 | 13.5 | 0.337 | -0.010 | -0.019 | -0.001 | 0.023 | -0.013 | -0.026 | 0.000 | 0.044 | 0.438 |
| UKB | Right leg fat percentage | 13 | 14.0 | 0.303 | -0.008 | -0.017 | 0.001 | 0.086 | -0.012 | -0.026 | 0.001 | 0.072 | 0.374 |
| PMID: 28743860 | Pediatric bone mineral density | 8 | 6.4 | 0.494 | 0.077 | -0.054 | 0.207 | 0.249 | -0.101 | -0.292 | 0.090 | 0.300 | 0.082 |
| PMID: 28743860 | Lean body mass | 8 | 2.3 | 0.939 | 0.115 | -0.009 | 0.240 | 0.069 | 0.098 | -0.102 | 0.298 | 0.335 | 0.801 |
| GEFOS | Whole body lean mass | 5 | 3.1 | 0.535 | -0.077 | -0.427 | 0.273 | 0.667 | -0.199 | -0.766 | 0.367 | 0.490 | 0.699 |
| GEFOS | Appendicular lean mass | 5 | 2.7 | 0.603 | 0.016 | -0.179 | 0.211 | 0.874 | -0.092 | -0.423 | 0.239 | 0.585 | 0.408 |
| FinnGen | Diseases of the musculoskeletal system and connective tissue | 13 | 17.8 | 0.119 | 1.006 | 0.963 | 1.051 | 0.797 | 0.950 | 0.902 | 1.001 | 0.051 | 0.003 |
| FinnGen | Soft tissue disorders | 13 | 8.53 | 0.742 | 0.962 | 0.920 | 1.007 | 0.095 | 0.940 | 0.888 | 0.996 | 0.035 | 0.043 |
| UKB | Knee osteoarthritis | 13 | 8.22 | 0.767 | 0.990 | 0.933 | 1.050 | 0.734 | 0.989 | 0.918 | 1.066 | 0.781 | 0.454 |
| UKB | Hip osteoarthritis | 13 | 21.5 | 0.042 | 0.976 | 0.901 | 1.058 | 0.552 | 1.011 | 0.888 | 1.152 | 0.868 | 0.427 |
| UKB | Hospital diagnosed osteoarthritis | 13 | 9.18 | 0.687 | 0.926 | 0.820 | 1.047 | 0.220 | 0.876 | 0.750 | 1.023 | 0.094 | 0.186 |
| FinnGen | Osteoporosis with pathological fracture | 13 | 17.2 | 0.141 | 0.858 | 0.631 | 1.168 | 0.330 | 0.984 | 0.604 | 1.602 | 0.947 | 0.918 |

CI, confidence interval; GEFOS, GEnetic Factors for OSteoporosis Consortium; The summary statistics data of UKB can be download from Nealelab (http://www.nealelab.is/uk-biobank); The summary statistics data in FinnGen Consortium can be downloaded from Google cloud storage free of charge. SNP, single nucleotide polymorphism; The Q statistic was used to present the heterogeneity among estimates for each SNPs in one analysis; For continuous outcomes, Effect means the value of Beta, and for categorical outcomes, Effect is the value of OR; The P value for the intercept in the MR-Egger regression was used present the pleiotropy (P < 0.05).

**Supplementary Table 7.** Associations of genetically predicted circulating homocysteine and vitamin B12 with fat and musculoskeletal phenotypes in the MR-PRESSO analysis.

| Source | Outcome | Homocysteine | | | | | | | | Vitamin B12 | | | | | | | |
| --- | --- | --- | --- | --- | --- | --- | --- | --- | --- | --- | --- | --- | --- | --- | --- | --- | --- |
|  |  | SNPs  used | Outliers | P_Glo | P_Dis | Effect | SE | | *P* | SNPs  used | Outliers | P_Glo | P_Dis | Effect | | SE | *P* |
| UKB | Body fat percentage | 14 | 4 | <0.001 | 0.492 | 0.014 | | 0.012 | 0.303 | 13 | 0 | 0.407 | NA | | -0.010 | 0.005 | **0.049** |
| UKB | Trunk fat percentage | 14 | 4 | <0.001 | 0.574 | 0.015 | | 0.016 | 0.394 | 13 | 0 | 0.352 | NA | | -0.012 | 0.006 | **0.049** |
| UKB | Left arm fat percentage | 14 | 3 | <0.001 | 0.455 | 0.029 | | 0.012 | 0.031 | 13 | 0 | 0.307 | NA | | -0.012 | 0.005 | **0.029** |
| UKB | Right arm fat percentage | 14 | 3 | <0.001 | 0.945 | 0.010 | | 0.015 | 0.510 | 13 | 0 | 0.375 | NA | | -0.011 | 0.005 | **0.044** |
| UKB | Left leg fat percentage | 14 | 3 | <0.001 | 0.589 | 0.014 | | 0.011 | 0.214 | 13 | 0 | 0.37 | NA | | -0.009 | 0.004 | **0.040** |
| UKB | Right leg fat percentage | 14 | 4 | <0.001 | 0.749 | 0.023 | | 0.008 | 0.022 | 13 | 0 | 0.332 | NA | | -0.008 | 0.004 | 0.098 |
| PMID: 28743860 | Pediatric bone mineral density | 13 | 0 | 0.564 | NA | -0.023 | | 0.052 | 0.662 | 8 | 0 | 0.466 | NA | | 0.042 | 0.050 | 0.432 |
| PMID: 28743860 | Lean body mass | 13 | 0 | 0.213 | NA | 0.015 | | 0.065 | 0.819 | 8 | 0 | 0.932 | NA | | 0.076 | 0.031 | 0.043 |
| GEFOS | Whole body lean mass | 13 | 0 | 0.127 | NA | 0.213 | | 0.160 | 0.207 | 5 | 0 | 0.545 | NA | | -0.104 | 0.134 | 0.481 |
| GEFOS | Appendicular lean mass | 13 | 0 | 0.102 | NA | 0.132 | | 0.100 | 0.211 | 5 | 0 | 0.613 | NA | | 0.027 | 0.073 | 0.727 |
| FinnGen | Diseases of the musculoskeletal system and connective tissue | 13 | 0 | 0.05 | NA | 1.076 | | 0.040 | 0.091 | 13 | 0 | 0.146 | NA | | 1.011 | 0.020 | 0.600 |
| FinnGen | Soft tissue disorders | 13 | 0 | 0.614 | NA | 1.069 | | 0.030 | **0.047** | 13 | 0 | 0.589 | NA | | 0.985 | 0.015 | 0.348 |
| UKB | Knee osteoarthritis | 14 | 0 | 0.11 | NA | 1.129 | | 0.047 | **0.022** | 13 | 0 | 0.712 | NA | | 0.967 | 0.019 | 0.112 |
| UKB | Hip osteoarthritis | 14 | 1 | 0.042 | 0.237 | 1.031 | | 0.057 | 0.604 | 13 | 1 | 0.041 | 0.087 | | 1.008 | 0.034 | 0.817 |
| UKB | Hospital diagnosed osteoarthritis | 14 | 0 | 0.823 | NA | 1.179 | | 0.063 | **0.022** | 13 | 0 | 0.687 | NA | | 0.951 | 0.043 | 0.265 |
| FinnGen | Osteoporosis with pathological fracture | 13 | 0 | 0.591 | NA | 1.603 | | 0.186 | **0.026** | 13 | 0 | 0.155 | NA | | 1.004 | 0.147 | 0.981 |

GEFOS, GEnetic Factors for OSteoporosis Consortium; The summary statistics data of UKB can be download from Nealelab (http://www.nealelab.is/uk-biobank); The summary statistics data in FinnGen Consortium can be downloaded from Google cloud storage free of charge. SE, standard error; SNP, single nucleotide polymorphism; NA, not available; For continuous outcomes, Effect means the value of Beta, and for categorical outcomes, Effect is the value of OR; P_Glo, p value for global test; P_Dis, p value for distortion test.

**Supplementary Table 8.** Validating the significant IVW results of homocysteine with soft tissue disorders, knee osteoarthritis, hospital diagnosed osteoarthritis, and osteoporosis with pathological fracture by using the Multivariable Mendelian randomization adjusting education attained, smoking and alcohol usage.

| Outcome | Samples | SNPs | OR | 95%CI | P |
| --- | --- | --- | --- | --- | --- |
| Soft tissue disorders | 218,792 | 13 | 1.064 | 0.997-1.137 | 0.063 |
| Knee osteoarthritis | 455,221 | 14 | 1.129 | 1.024-1.245 | 0.015 |
| Hospital diagnosed osteoarthritis | 327,918 | 14 | 1.172 | 1.003-1.368 | 0.045 |
| Osteoporosis with pathological fracture | 173,619 | 13 | 1.560 | 1.010-2.412 | 0.045 |

CI, confidence interval; SNP, single nucleotide polymorphism; OR, odds ratio; IVW, inverse variance weighted method.

**Supplementary Table 9.** Validating the significant IVW results of vitamin B12 with fat percentage by using the Multivariable Mendelian randomization adjusting education attained, smoking and alcohol usage.

| Outcome | Samples | SNPs | Beta | 95%CI | | P |
| --- | --- | --- | --- | --- | --- | --- |
| Body fat percentage | 331,117 | 13 | -0.009 | -0.019 | 1.17E-03 | 0.083 |
| Trunk fat percentage | 331,117 | 13 | -0.011 | -0.023 | 1.43E-03 | 0.083 |
| Arm fat percentage (left) | 331,117 | 13 | -0.012 | -0.023 | -1.29E-03 | 0.029 |
| Arm fat percentage (right) | 331,117 | 13 | -0.012 | -0.022 | -8.89E-04 | 0.034 |
| Leg fat percentage (left) | 331,117 | 13 | -0.008 | -0.016 | 7.57E-04 | 0.074 |
| Leg fat percentage (right) | 331,117 | 13 | -0.006 | -0.015 | 3.06E-03 | 0.198 |

CI, confidence interval; SNP, single nucleotide polymorphism; IVW, inverse variance weighted method.

**Supplementary Table 10.** The significant IVW results of vitamin B12 with fat percentage, homocysteine with soft tissue disorders, knee osteoarthritis, hospital diagnosed osteoarthritis, and osteoporosis with pathological fracture are validated for the possible reverse associations via the reverse Mendelian randomization analysis.

| Outcome | Exposure | Samples | Effect | 95%CI | | P |
| --- | --- | --- | --- | --- | --- | --- |
| Vitamin B12 | Body fat percentage | 331,117 | -1.728 | -4.079 | 0.621 | 0.149 |
|  | Trunk fat percentage | 331,117 | -1.649 | -3.587 | 0.288 | 0.095 |
|  | Arm fat percentage (left) | 331,117 | -1.710 | -3.560 | 0.138 | 0.069 |
|  | Arm fat percentage (right) | 331,117 | -1.777 | -3.768 | 0.213 | 0.080 |
|  | Leg fat percentage (left) | 331,117 | -1.860 | -4.271 | 0.550 | 0.130 |
|  | Leg fat percentage (right) | 331,117 | -1.339 | -3.792 | 0.111 | 0.284 |
| Homocysteine | Soft tissue disorders | 218,792 | 0.373 | -0.191 | 0.939 | 0.195 |
|  | Knee osteoarthritis | 455,221 | 0.216 | -0.072 | 0.505 | 0.142 |
|  | Hospital diagnosed osteoarthritis | 327,918 | 0.132 | -0.107 | 0.373 | 0.278 |
|  | Osteoporosis with pathological fracture | 173,619 | 0.083 | -4.42E-06 | 0.166 | 0.051 |

CI, confidence interval; IVW, inverse variance weighted method.
